# Minimizing Thermal Stress for Data Center Servers through Thermal-Aware Relocation

**DOI:** 10.1155/2014/684501

**Published:** 2014-03-31

**Authors:** Muhammad Tayyab Chaudhry, T. C. Ling, S. A. Hussain, Atif Manzoor

**Affiliations:** ^1^Universiti Malaya, 50603 Kuala Lumpur, Wilayah Persekutuan, Malaysia; ^2^COMSATS Institute of IT, 54000 Lahore, Punjab, Pakistan

## Abstract

A rise in inlet air temperature may lower the rate of heat dissipation from air cooled computing servers. This introduces a thermal stress to these servers. As a result, the poorly cooled active servers will start conducting heat to the neighboring servers and giving rise to hotspot regions of thermal stress, inside the data center. As a result, the physical hardware of these servers may fail, thus causing performance loss, monetary loss, and higher energy consumption for cooling mechanism. In order to minimize these situations, this paper performs the profiling of inlet temperature sensitivity (ITS) and defines the optimum location for each server to minimize the chances of creating a thermal hotspot and thermal stress. Based upon novel ITS analysis, a thermal state monitoring and server relocation algorithm for data centers is being proposed. The contribution of this paper is bringing the peak outlet temperatures of the relocated servers closer to average outlet temperature by over 5 times, lowering the average peak outlet temperature by 3.5% and minimizing the thermal stress.

## 1. Introduction

With the rapid proliferation of cloud services, the data center servers are experiencing increasing computational load each year. The electrical power consumed by IT equipment is converted into heat [1]. An equal amount of power is required to remove that heat in order to maintain a proper working environment via cooling mechanism. The cooling mechanism works by blowing the cold air through hollow floor tiles towards server racks. As a natural process, the temperature of cold air blown from the floor vents becomes more than the set temperature near the top of the racks. In addition to that, the hot air blown out from the air cooled servers from the back of the racks rises up and gets mixed with the cold air near the top of the racks. This recirculation of heat increases the cold air temperature as well [2–4]. Thus, the top mounted rack servers become the victims of inlet temperature increment.

In a server which is a victim of high inlet temperature, the heat removal efficiency is reduced. Particularly, when servers are generating maximum heat at full utilization, the hardware experiences thermal strain which changes to thermal stress [5]. These poorly cooled servers start conducting heat to neighboring servers causing them to become undercooled. Over a period of time, the heat generated in the undercooled servers may exceed the rate of dissipation and a hotspot is formed. Hotspots lead to hardware failure as well as performance loss and violation of service level agreement (SLA). In addition to this, a hotspot detected by data center thermal monitoring system may trigger the cooling mechanism to cool down the hotspot, thus leading to increased total cost of ownership [6] of data center.

Heat dissipated by the servers depends upon their utilization levels and power consumption and can be marked by their outlet temperatures. Heterogeneous servers dissipate different amount of heat at same level of utilization and power consumption. This can be verified from the power consumption and heat dissipation statistics of the processors as well. The variation in inlet temperature has the typical effect over heat dissipation of servers that can be profiled for inlet temperature sensitivity (ITS). This paper demonstrates that the hotspots can be minimized if the servers are placed according to ITS profiling analysis. Each server undergoes a thermal state transition on the basis of inlet temperature variations. Hotspot is the extreme thermal state which lays thermal stress over servers. The servers inside hotspots can be relocated on the basis of same similar analysis to reduce the reoccurring of hotspots and to minimize thermal stress.

This paper is organized as follows.  Section 2 shows the related literature review.  Section 3 introduces the concepts and terminology used in rest of the paper.  Section 4 describes the ITS analysis with respect to thermal state transition and also describes the thermal-aware relocation of data center servers. The experimental results and discussion are covered by Section 5. Finally, the conclusion is presented in Section 6.

## 2. Related Work

Thermal modeling and temperature estimation [7, 8] from thermal sensors should consider that the increase in inlet air temperature may cause some servers to undergo hotspot conditions and thermal stress. This is because they are not placed at proper positions according to thermal-aware location analysis. Thermal-aware server provisioning approach with the objective of minimizing the total power consumption of data center [4, 9] calculates the power by considering the maximum working temperature of the servers. Such calculation should also consider that the inlet temperature rise may cause the servers to reach to the maximum temperature and cause thermal stress.

Computational fluid dynamics (CFD) is popular tool for engineers. Workload placement techniques that rely upon CFD simulations [2, 4, 10] can give the estimation of thermal stress besides the data center power consumption for cooling and computing, if the location of servers, the inlet temperature variation, and thermal-stress phenomenon are included in the respective energy models. A technique to reduce recirculation of hot air inside data center [2] can perform better and save more cooling energy if the recirculation of hot air is distinguished from the natural heating-up of cold air after it is blown from vent tiles. However, the factor of reliability and thermal stress due to heat recirculation should also be considered.

The thermal data gathered from a range of thermal sensors will have the noise of acquired heat in cold air [11, 12] due to physical phenomenon and/or through heat recirculation. If this data is used for cooling control, such as implementing ASHRAE [13] standards, then the servers should be placed according to their thermal sensitivity to inlet temperature before data gathering could begin. Since the ASHRAE [13] requires the data center cooling temperature to be increased, doing so across data center will put some servers to go under thermal stress due to heat recirculation. Therefore, before making any decision regarding a raise in cooling temperature, the data center management should perform a thermal-stress evaluation for data center servers according to their location and inlet temperature.

The coefficients of heat recirculation and heat extraction for the data center servers [12] are sensitive to the inlet temperature increment and the value of coefficients should not be affected by this phenomenon. The data center workload scheduling techniques by RC-thermal model of heat exchange [14, 15] should consider that the backfilling of workload may not work well if the change in inlet temperature is not considered. Additionally, the backfilling can cause hotspots and thermal stress upon the serves located in high inlet temperature region of data center. Task-temperature profiles used for thermal-aware workload scheduling should consider the effect of inlet temperature sensitivity of the physical servers upon the scheduling outcome in terms of thermal map to be unexpected [16].

The importance of arranging the servers according to thermal-stress analysis is that a thermal-aware workload scheduling algorithm to consolidate active servers [17] will have low chances of creating hotspots. Similarly if the power profiles of servers are made as in [18], then they will have less errors if the profiling is performed after performing the server arrangement for minimized thermal stress. If the power saving techniques such as diskless booting [19] are used, then the servers will dissipate even less heat and undergo a minimum thermal stress if they are located in a thermal-aware arrangement.

If the power consumption profiles of server are created so that the least power is used to execute a given computing load and to ensure performance and profit as in [20], then the scheduling algorithm can save more power if the hotspots are avoided. Additionally, the monitory loss due to hardware failure can be avoided if the servers undergo minimum thermal stress. The thermal profiling based techniques [21, 22] cannot give accurate results unless it is assured that the servers are efficiently placed across the data center in thermal-aware manner as proposed in this paper. In order to achieve a high thermostat setting for air conditioning [3, 23], the proper placement of servers at optimum positions should be prerequisite before evaluating the power consumption of data center. Raising the cold air temperature can save cooling power but it will increase thermal stress for the servers affected by heat recirculation. Eventually those servers at high utilization will experience thermal stress and may fail while the cooling mechanism might also be using more energy to cool down the hotspots.

The data center power management and server consolidation techniques can avoid hotspots, thermal stress, and unnecessary power usage for cooling by placing the servers at the most optimum positions according to thermal-stress analysis. Scheduling algorithms to minimize heat can have improved performance if the servers are placed at optimum location to minimize thermal stress. A server may undergo various thermal states according to different factors such as thermal stress, computational load, and inlet air temperature. By identifying the thermal states of each server, an optimum location can be identified according to balance of these factors and to minimum of the thermal stress. This paper presents thermal state modeling approach for data center servers to identify and minimize the thermal stress through server relocation. The benefits are reduced thermal stress, minimum hotspots, and cooling energy saving in data centers.

## 3. Background

By the law of energy conservation, the watts of electrical power consumed are converted into equivalent joules of thermal energy [1]. If *E*_computing_^*i*^ is the electricity consumed by a data center server *i*, then this energy is converted to *E*_Joules_^*i*^:

(1) $$\begin{array}{c} E_{\text{computing}}^{i}=E_{\text{Joules}}^{i}.\; \end{array}$$

The air gets less cold when it travels towards servers after being blown from the perforated tiles of hollow floor. The hike in inlet air temperature, due to this and recirculation of heat, has a direct impact over outlet air temperature for each server. So the outlet air temperature rises by the rise in inlet temperature. But this relation is not strictly linear as the rise in inlet air temperature makes it a weaker conductor of heat. The rate of heat transfer *Q* ^*i*^ from server *i* by conduction through air [24] is given by

(2) $$\begin{array}{c} Q^{i}=k^{i}A^{i}\left(\;T_{\text{server}}^{i}-T_{\text{received}}^{i}\right), \end{array}$$

where *k* ^*i*^ is the coefficient of heat transfer for server *i*, *A* ^*i*^ is the overall area inside server through which the cold air at temperature *T*_received_^*i*^ flows and cools the server through conduction, and *T*_server_^*i*^ is the overall temperature of the hardware inside server casing. The rise in inlet air temperature slows down the rate of heat transfer from the server depending upon the make and model of the server. The coefficient *k* ^*i*^ may be different for heterogeneous servers. The coefficient of performance (COP) curve [25] is unable to give a solution to the situation when a server is getting hot due to rise in inlet air temperature. The server having high temperature of inlet air *T*_received_ will have a corresponding increase in the outlet air temperature as shown below

(3) $$\begin{array}{c} {\Delta T}^{i}=T_{\text{received}}^{i}-T_{\text{set}}, \end{array}$$

where Δ*T* ^*i*^ is the increase in inlet temperature of server *i*. The highly dense arrangement of bare bone blade servers [26] can hold up to 96 servers in 45 u rack space. Such a dense existence of server can suffer fatal thermal stress when a server *i* is exposed to increased inlet air temperature Δ*T* ^*i*^ as shown in (3). The thermal stress *σ* [27] can be represented as follows:

(4) $$\begin{array}{c} \sigma^{i}=E^{\prime }\alpha^{i}\Delta T^{i}, \end{array}$$

where *E*′ is the modulus of elasticity of the server *i* and *α* ^*i*^ is the coefficient of thermal expansion in m/m°C for server *i*. The increase in inlet temperature causes thermal stress. Over a period of time, this may eventually cause hardware failure as the servers are tightly mounted in racks. The increased outlet temperature *T*_outle(increased)_^*i*^ of a server due to increase in inlet temperature has three effects.

- First, it puts extra burden on cooling mechanism as the outlet temperature of the servers is increased.
- Secondly, it may cause hotspot.
- It may lay thermal-stress over server hardware.

Data center servers have a built-in mechanism to dynamically adjust the outlet fan rotation with respect to inlet temperature as a reactive thermal management technique. This is to increase the airflow inside server casing to maintain heat flow and to reduce the thermal stress. The dynamic fan rotation control may lead to loud noise and/or hardware damage if the fan gets damaged [13]. Some dynamic thermal management (DTM) routines apply frequency scaling to lower down processor speed in order to cool it down. Disabling the dynamic fan control requires the inlet temperature to be within a vendor specified maximum value *T*_max⁡⁡inlet_^*i*^.

Consider a maximum threshold outlet temperature *T*_threshold_ from a server that is marked as hotspot temperature by the monitoring system. A subthreshold (*T*_max⁡⁡inlet_^*i*^ − *β*) is defined for the indication of thermal stress. The value for *β* is numeric and depends on COP.

In highly dense arrangement of modern day blade servers, a rise in inlet air temperature followed by a rise in computational load will put the servers in thermal-stress state. This will not only result in hotspots and equipment failure, but also increase the data center wide cooling energy consumption. In particular, at inlet temperature between (*T*_max⁡⁡inlet_^*i*^ − *β*) and *T*_max⁡⁡inlet_^*i*^, the server *i* at high utilization will start experiencing thermal stress because DTM will be inactive. In this situation, the outlet temperature of a fully utilized server is maximized and may exceed the peak temperature threshold *T*_threshold_. At this time, a hotspot is initiated by server *i*. It is important to lower down the peak outlet temperature of server *i* to avoid hotspot. To lower the peak outlet temperature, it is better to relocate the server instead of shifting the workload. Server relocation provides a permanent solution to hotspots.

## 4. Algorithm for Thermal-Aware Server Relocation to Minimize Thermal Stress

This section presents the algorithm to reduce the thermal stress through thermal-aware server relocation, those servers which are/were part of hotspot and those which are likely to initiate hotspots are considered. This paper is proposed to make a thermal profile of all the data center servers with respect to inlet temperature. Inlet temperature effect (ITE) thermal benchmark test can be used for this purpose. ITE test reveals the change in outlet temperature of a server with respect to changes in inlet temperature at zero and full CPU utilization levels. (In the rest of this paper, the phrase server utilization refers to CPU utilization because CPU is the most power consuming and the most heat dissipating hardware component of any computer system.) The values of *T*_max⁡⁡inlet_^*i*^ and *T*_outle(increased)_^*i*^ can be inferred from ITE test. Homogenous servers have the same *T*_max⁡⁡inlet_^*i*^. However, *T*_outle(increased)_^*i*^ depends upon the location of temperature monitoring sensors and can be verified with multiple tests with different sensor locations.

### 4.1. Thermal State Transition

**Figure 1:**
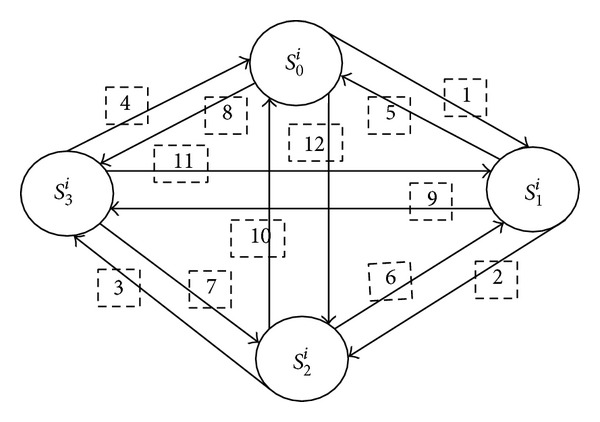
State transition diagram for data center server.

A finite set of thermal states for a server *i* inside data center is demonstrated in the section with respect to inlet temperature, outlet temperature, server utilization level, and thermal stress. A thermal state of a server *i* can be defined as a tuple (*T*_inlet_^*i*^, *T*_outlet_^*i*^, *μ*, *σ* ^*i*^) and represented by a notation *S*_*n*_^*i*^, where *n* is a whole number and has a range from 0 to 3 as per the state transition diagram demonstrated in Figure 1. The domains of the elements of thermal states are defined in Table 1. It is assumed that for all the states *T*_received_^*i*^ < *T*_threshold_. The states *S*_0_^*i*^ and *S*_1_^*i*^ are the desired states where there is no thermal stress. State *S*_0_^*i*^ is the idle state where the server has no workload *μ* and the inlet temperature is close to the set temperature *T*_set_. State *S*_1_^*i*^ is an active state of the server where *μ* is not idle and the inlet temperature is same as that of *S*_0_^*i*^. Both these initial states have outlet temperature below the red line temperature *T*_threshold_.

The difference between states *S*_2_^*i*^ and *S*_3_^*i*^ is that the formal is an indicator of future thermal stress and future hotspots. State *S*_3_^*i*^ may have thermal stress due to higher inlet temperature compared to state *S*_2_^*i*^. State *S*_3_^*i*^ is the hotspot state with outlet temperature being more than the maximum threshold and the presence of thermal stress.

Figure 1 demonstrates the state transition diagram where all the states are at mesh. The conditions for state transition are given in Table 2. The desirable states are *S*_0_^*i*^ to *S*_1_^*i*^ on the basis of server utilization at Δ*T* ^*i*^ equal to zero or minimum. When Δ*T* ^*i*^ becomes significant but remains lower than subthreshold (*T*_max⁡⁡inlet_^*i*^ − *β*) at any state, the yellow marked *S*_2_^*i*^ is reached. This state is an indication of future thermal-stress and likelihood of hotspot. For any active server, the violation of (*T*_max⁡⁡inlet_^*i*^ − *β*) subthreshold represented by ((*T*_max⁡⁡inlet_^*i*^ − *β*) − *T*_received_^*i*^) ≤ 0, at any state, makes the respective server reach to hotspot state *S*_3_^*i*^. An idle server with this subthreshold violation is considered in state *S*_0_^*i*^ if the outlet temperature is below *T*_threshold_. By following the relocation algorithm presented in next subsection, the servers from state *S*_3_^*i*^ can be brought to lower states and thermalstress can be removed.

This paper defines thermal profiling process consisting of noting down the outlet temperature at minimum and maximum utilization of server when the inlet temperature is stable and below DTM threshold. For each server *i*, a thermal profile can be defined as a tuple having three elements: *T*_max⁡⁡inlet_^*k*^, *t*_min⁡⁡CPU_^*i*^, and *t*_max⁡⁡CPU_^*i*^ where the second and third elements are equal to *T*_outlet_^*i*^ − *T*_received_^*i*^ at minimum and maximum CPU utilization, respectively. The difference between *t*_min⁡⁡CPU_^*i*^ and *t*_max⁡⁡CPU_^*i*^ shows the typical value of maximum increase in outlet temperature for any server when *T*_received_^*i*^ < *T*_max⁡⁡inlet_^*i*^.

### 4.2. Thermal-Aware Server Relocation Algorithm for Minimizing Thermal Stress

For each server at state *S*_3_^*i*^ the relocation algorithm can be given as in Algorithm 1.

The algorithm searches for a suitable server from the set of server in state *S*_0_^*i*^ which can withstand the high inlet temperature (listing (3)). Alternatively, a server in state *S*_1_^*i*^ is searched with more strict criteria that the maximum outlet temperature is below the hotspot server in addition to the inlet maximum temperature check (listing (8-9)). This is to make sure there will be no reoccurring hotspot after switching. In case no server is found in lower states, the higher state *S*_2_^*i*^ servers are searched with most strict criteria that the minimum CPU outlet of *S*_2_^*i*^ server is lower than the hotspot server (listing (14–16)). In case there is no suitable server for location switching, in the entire data center, the algorithm suggests shifting the workload from hotspot server to a server in state *S*_0_^*i*^ (listing (20)). Thus the proposed algorithm can minimize the chances of hotspot of the servers in state *S*_3_^*i*^. The next section demonstrates the effectiveness of location switching.

## 5. Experimental Setup

The proposed approach was tested over a set of heterogeneous servers of make HP Paviolion ML350 G5. The servers have VMware ESXi 5.0 [28] hypervisor installed. Virtualized servers (hosts) were used because the virtualization has a wide scope for cloud computing and virtualized data centers. The servers are classified as type A and type B. Type A has two quad core Intel(R) Xeon(R) CPU E5430 2.66 GHz processors. Type B has Intel(R) Xeon(R) CPU E5320 1.86 GHz processor. Each server has 6 GB of RAM. A set of 8 virtual machines (VMs) was executed on each server during the experiments. All VMs were single virtual CPU (vCPU) with maximum vCPU (limit kept in suspended state) with CPU intensive benchmark Prime95 [29] running over each VM when suspended. Thermal stress was introduced by thermostat settings of the air condition unit. BY raising the set temperature, a scenario of heat recirculation was created during the experiments.

To monitor the inlet and outlet air temperatures, external USB thermal sensors were used. The power consumption of each host was measured by USB smart power meters. Microsoft C# script was used to manipulate the VM operations such as powering on VMs with a specified batch size and VM suspension. The servers are placed inside research lab room with dimensions of 25 feet × 30 feet. There are total 10 desktops and 2 servers inside lab. Two of the desktops are Intel corei7 while the other 8 are Intel Pentium4. Each desktop has a standard size LCD. There are two network switches and two wireless routers. There are two split air conditioners inside lab with 2-ton cooling capacity each. The servers are placed under the table about 10 feet away from air conditioners. The tables are arranged horizontal to the airflow of air conditioners.

### 5.1. Experimental Results and Discussion

**Figure 2:**
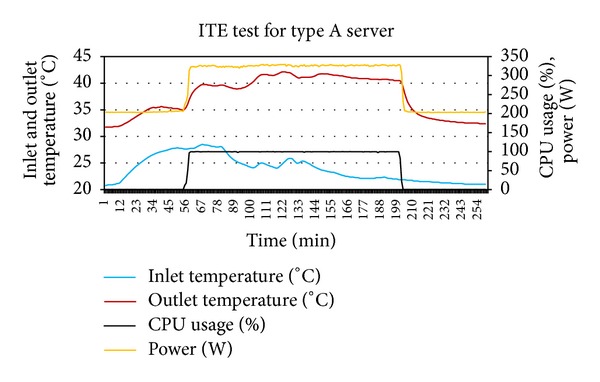
ITE test performed for type A server.

**Figure 3:**
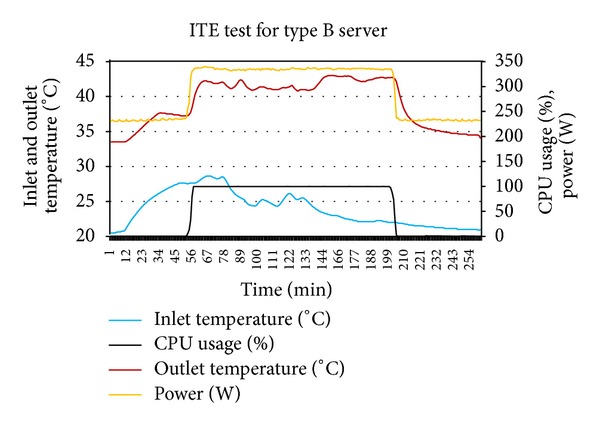
ITE test performed for type B server.

As a first step, ITE test was performed by varying inlet temperature of the servers through thermostat setting at minimum and maximum utilization of servers. Figures 2 and 3 show the output of experiment. The thermal variables gathered from the ITE tests are shown in Table 3.

The maximum inlet temperature for both servers was set at 23 Celsius on the basis of results of ITE test as shown in Table 3. In order to profile the servers for *t*_max⁡⁡CPU_^*i*^, the step linear increment (SLI) test was performed. In this test, the CPU intensive workload is put over servers in steps, where each step involves the powering on of one VM after a fixed interval of time such that the last step brings the CPU utilization of the host to maximum. The inlet temperature is kept stable by placing the servers at a proper location. Such locations were found by placing thermal sensors around the research lab and the readings were observed over few days to mark the suitable regions of room with stable temperature.

**Figure 4:**
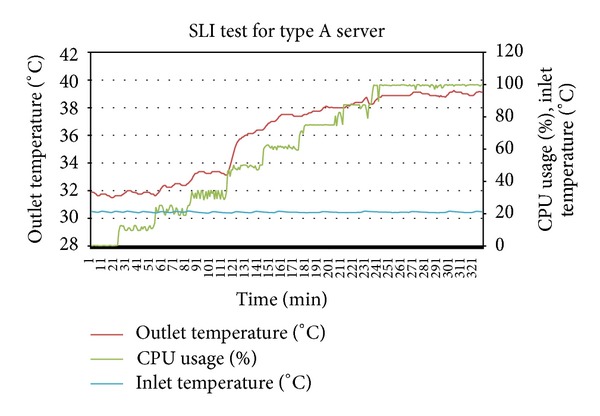
SLI test performed for thermal profiling of type A server.

**Figure 5:**
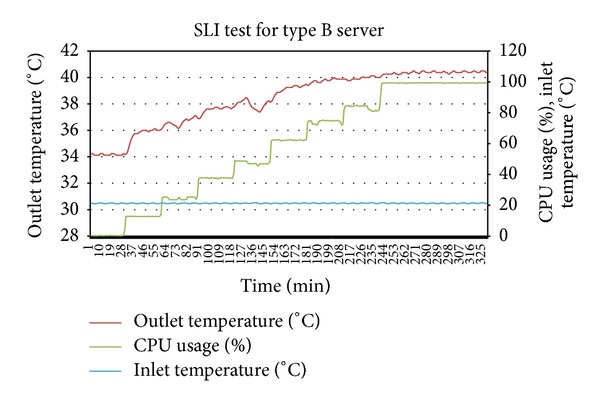
SLI test performed for thermal profiling of type B server.

Thermal profiles were created from two SLI tests at different but stable inlet temperatures. For first SLI test, the average temperature was 21.3 Celsius which was well below value of (*T*_max⁡⁡inlet_^*i*^ − *β*) subthreshold. The test results are shown in Figures 4 and 5.

**Figure 6:**
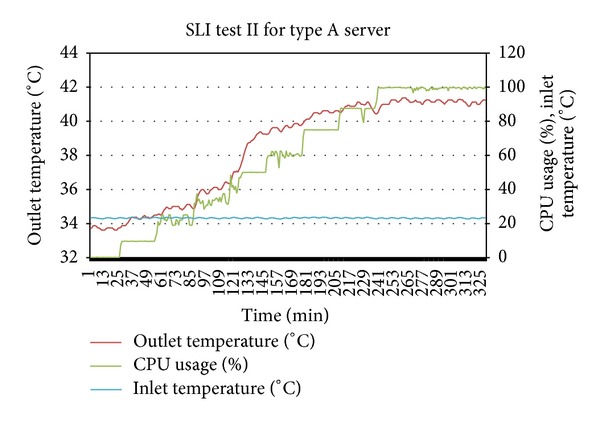
SLI test for server type A for state determination and thermal profile verification.

**Figure 7:**
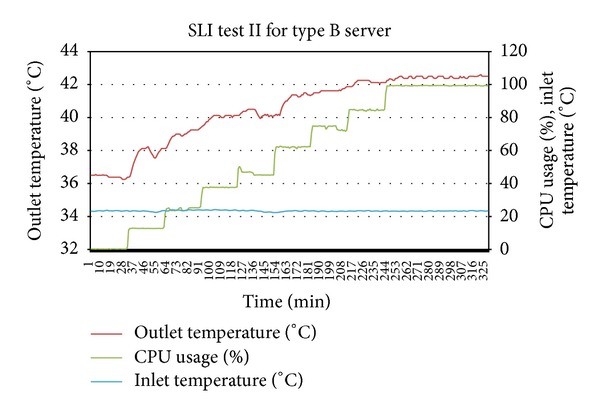
SLI test for server type B for state determination and thermal profile verification.

For the second SLI test, the average inlet temperature was 23.5 Celsius which means that it is a hotspot causing inlet temperature given by (*T*_max⁡⁡inlet_^*i*^ − *β*) − *T*_received_^*i*^ ≤ 0. Thermal profiles for the servers are shown in Table 4, while the test results are shown in Figures 6 and 7. If the inlet temperature remains stable and below the threshold of DTM, a thermal profile for the servers can be created. In this paper, the thermal profiles were created by SLI experiments of Figures 4 and 5 and then verified later at hotspot causing inlet temperature in Figures 6 and 7. The results show that the outlet temperature of a server can be predicted by extrapolation and interpolation of outlet temperatures with respect to increase and decrease in inlet temperature, respectively. This paper verifies that the average peak outlet temperatures of the prototype servers can be extrapolated within a range of average inlet temperature range 21.3–23.5 Celsius. The detailed results are available at [25].

#### 5.1.1. Evaluation of State Transition Diagram

By putting the *T*_threshold_ value to 42 Celsius and using the thermal profiles of the prototype servers, the occurrence of hotspot and thermal stress can be verified. Considering the servers in Figures 4 and 5 were in states *S*_0_^*i*^ and *S*_1_^*i*^ according to inlet temperature, then the servers were exposed to hotspot and thermal stress causing inlet temperature in Figures 6 and 7. Comparison of both sets of SLI test results shows that the type B server can reach to state *S*_3_^*i*^ as per thermal state transition diagram of Figure 1. Type B server can be considered under thermal stress as average Δ*T* ^*i*^ = 0.5 Celsius.

Consider the server relocation algorithm by supposing that type B server is active and has inlet temperature violating subthreshold (*T*_max⁡⁡inlet_^*i*^ − *β*) and the state of that server is *S*_3_^*i*^. Also suppose that inlet temperature of type A server is below subthreshold (*T*_max⁡⁡inlet_^*i*^ − *β*) and the server is in state *S*_1_^*i*^. Following the location switching algorithm, the type B server can be relocated by location switching with type A server. So after relocation, both servers are at state/s lower than *S*_3_^*i*^. This will also proves that the relocated servers will have

- homogenous outlet temperature despite different inlet temperatures;
- no hotspot;
- cooling cost saving.

**Figure 8:**
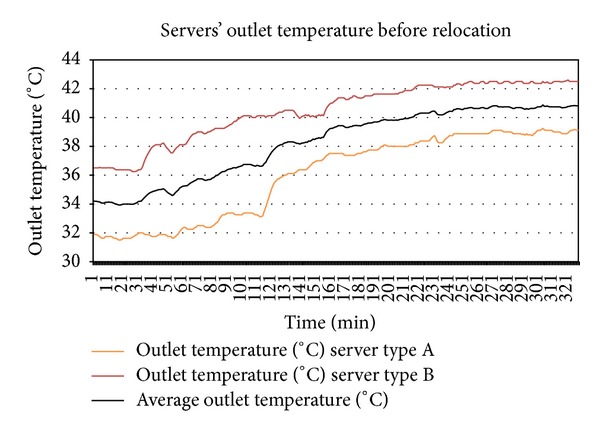
The outlet temperatures from SLI tests of servers before relocation.

**Figure 9:**
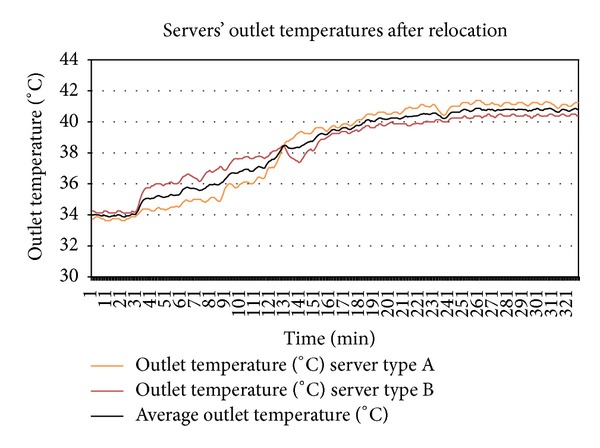
Outlet temperatures from SLI tests of the servers are more homogenous and close to average after relocation.

Plotting together the outlet temperatures of type A server from Figure 4 and of type B server from Figure 7 into Figure 8 shows that the outlet temperatures of servers are quite far from each other at all levels of utilization. Focus on the average peak outlet temperatures which are 3.5 Celsius apart. Before relocation, as shown in Table 4, the outlet temperatures of both servers were almost the same ±different from the average peak of outlet temperatures. If the servers are relocated, the immediate effect is the homogeneity of outlet temperatures at all levels of utilization, especially at the peak and idle states. By plotting the outlet temperatures of both servers form Figures 5 and 6 together in Figure 9, it can be observed that the outlet temperatures of both servers are closer to average temperature curve. Thus, the servers can be relocated by using thermal profiles as one of the inputs parameter to relocation algorithm.

Cooling cost is saved because all the relocated servers are not in state *S*_3_^*i*^ to trigger the cooling system and the peak average outlet temperature is reduced by 3.5% after relocation. As demonstrated in Table 5, there is a significant 5 to 7.65 times improvement in homogeneity of the average peak outlet temperatures of the servers after relocation. Both the servers are well below the maximum threshold of 42 Celsius after relocation. As a future work, detailed thermal profiles will be created and an outlet temperature prediction technique will be proposed on the basis of thermal profiles.

## 6. Conclusion

This paper showed that the data center servers undergo state transition from normal to thermal stress as a result of change in inlet temperature. The servers can be profiled with respect to ITS and outlet temperatures can be predicted from interpolation and extrapolation of thermal profiles. This will be presented in more detail in future work. The novel state transition and ITS analysis for servers presented in this paper manage to predict and track the state of a server when there is a change in inlet air temperature. This is a novel paper in which virtualized servers were used for thermal-stress and hotspot state evaluation. The servers inside hotspot area can be relocated on the basis of ITS profiling and thermal states based relocation algorithm presented in this paper. The algorithm can identify a more suitable location with minimum thermal stress for the hotspot affected servers. This relocation process will avoid hotspots, ensuring homogenous outlet temperatures across the data center, minimizing thermal stress, lowering the peak average outlet temperature, and saving cooling power. This paper shows that the peak average temperature was reduced by 3.5% and the peak outlet temperatures of the relocated servers were closer to the average by over 5 times. These results help in the establishment of thermal-stress free, green data centers.

## Figures and Tables

**Algorithm 1:**
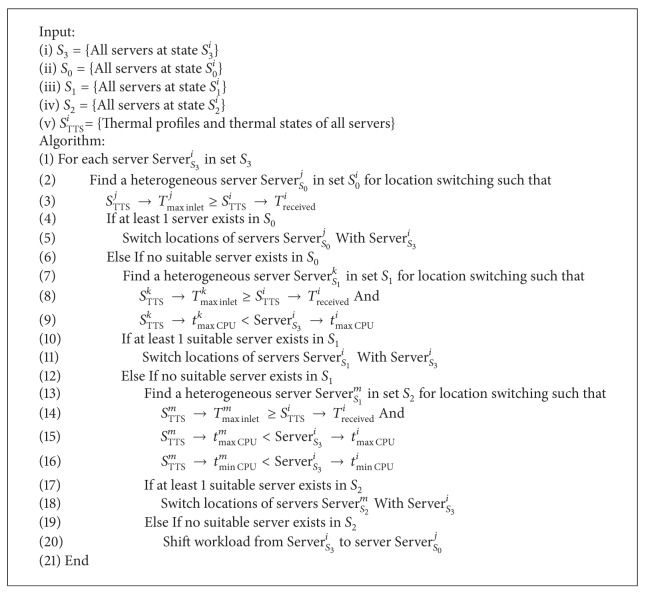


**Table 1:** Domain table for thermal states.

Thermal state	*T*_inlet_^*i*^	*T*_outlet_^*i*^	*μ*	*σ* ^*i*^	Chances of hotspot
*S*_0_^*i*^	*T*_received_^*i*^ ≈ *T*_set_	*T*_outlet_^*i*^ < *T*_threshold_	*≈*0	*≈*0	Nil
*S*_1_^*i*^	*T*_received_^*i*^ ≈ *T*_set_	*T*_outlet_^*i*^ < *T*_threshold_	>0	*≈*0	Nil
*S*_2_^*i*^	*T*_received_^*i*^ < (*T*_max⁡inlet_^*i*^ − *β*)	*T*_outlet_^*i*^ ≤ *T*_threshold_	>0	*≈*0	Yes
*S*_3_^*i*^	((*T*_max⁡inlet_^*i*^ − *β*) − *T*_received_^*i*^) ≤ 0	*T*_outlet_^*i*^ ≥ *T*_threshold_	>0	>0	Yes

**Table 2:** Thermal states transition conditions table.

Transition number	Condition/s
1	*μ* > 0
2	Δ*T* ^*i*^ > 0
3	{((*T*_max⁡inlet_^*i*^ − *β*) − *T*_received_^*i*^) ≤ 0} or (*T*_outlet_^*i*^ ≥ *T*_threshold_)
4	*μ* ≈ 0 | (Relocation and *μ* ≈ 0)
5	*μ* ≈ 0
6	Δ*T* ^*i*^ ≈ 0
7	Relocation and (Δ*T* ^*i*^ ≈ 0)
8	{((*T*_max⁡inlet_^*i*^ − *β*) − *T*_received_^*i*^) ≤ 0} and (*μ* > 0)
9	{((*T*_max⁡inlet_^*i*^ − *β*) − *T*_received_^*i*^) ≤ 0}
10	(Δ*T* ^*i*^ ≈ 0) and (*μ* ≈ 0)
11	Relocation and (Δ*T* ^*i*^ ≈ 0)
12	(Δ*T* ^*i*^ > 0) and (*μ* > 0)

**Table 3:** Thermal variables gathered from ITE test.

Server type	Thermal variable	Value (Celsius)
A	*T*_max⁡inlet_^A^	26 to 27
B	*T*_max⁡inlet_^B^	25 to 26
A	*β*	3
B	*β*	2
A	Average *t*_min⁡⁡CPU_^A^	11
B	Average *t*_min⁡⁡CPU_^B^	13

**Table 4:** Thermal profile elements calculated from SLI tests.

Server type	Thermal variable	Value (Celsius)
A	Average *t*_max⁡⁡CPU_^A^	18
B	Average *t*_max⁡⁡CPU_^B^	19
A	Average *t*_max⁡⁡CPU_^A^ − Average *t*_min⁡⁡CPU_^A^	7
B	Average *t*_max⁡⁡CPU_^B^ − Average *t*_min⁡⁡CPU_^B^	6

**Table 5:** Average outlet temperatures of relocated servers before and after relocation.

Before/after relocation	Peak average temperature server type A (Celsius)	Peak average temperature server type B (Celsius)	Overall average peak temperature (Celsius)	Difference from average peak temperature (Celsius) server type A	Difference from average peak temperature (Celsius) server type B
Before	39	42.5	40.7	−1.76	+1.74
After	41	40.4	40.7	+0.23	−0.35
% Change	5% increase	5% decrease	No change	765% improved	500% improved
